# No impact of Asian ethnicity on EORTC QLQ-C30 scores: Group differences and differential item functioning in paroxysmal nocturnal hemoglobinuria

**DOI:** 10.1186/s12955-021-01860-3

**Published:** 2021-09-28

**Authors:** Carolyn E. Schwartz, Roland B. Stark, Katrina Borowiec, Karl-Johan Myren

**Affiliations:** 1 DeltaQuest Foundation, Inc, 31 Mitchell Road, Concord, MA 01742 USA; 2 Departments of Medicine and Orthopaedic Surgery, Tufts University Medical School, Boston, MA USA; 3 Department of Measurement, Evaluation, Statistics, & Assessment, Boston College Lynch School of Education and Human Development, Chestnut Hill, MA USA; 4 Health Economics and Outcome Research, Alexion Pharmaceuticals, Inc, Stockholm, Sweden

**Keywords:** Quality of life, Paroxysmal nocturnal hemoglobinuria, Ethnicity, EORTC, Norms

## Abstract

**Background:**

Paroxysmal nocturnal hemoglobinuria (PNH) is a rare, life-threatening terminal-complement-mediated disease resulting in intravascular hemolysis and thrombosis with significant morbidity and premature mortality. There exists no disease-specific quality-of-life (QOL) measure for PNH. Its QOL effects resemble those of hematologic cancers, which supports the use of cancer-specific QOL measures in PNH clinical trials. The European Organisation for Research and Treatment of Cancer (EORTC) QLQ-C30 has published norms for many European and North American countries, but not for Asian countries. We investigated differences by Asian ethnicity in scores and item function on the EORTC QLQ-C30.

**Methods:**

This secondary analysis focused on two non-inferiority PNH trials (301 and 302) comparing eculizumab and ravulizumab (n = 441). Analysis of covariance examined the main effect of Asian ethnicity on baseline EORTC QLQ-C30 scores, after adjusting for propensity scores encompassing trial, demographic and clinical factors. Mixed modeling of longitudinal data compared subscale scores in Asian vs. non-Asian patients, after propensity adjustment. Differential item function (DIF) was examined using ordinal regression models at baseline and longitudinally, to predict item score from total score, ethnicity, and their interaction to test for uniform DIF (significant main effect for Asian) and non-uniform DIF (significant Asian-by-total-score interaction).

**Results:**

Of the 15 baseline domains, Asian patients scored slightly better on role and emotional functioning and slightly worse on constipation and diarrhea (0.22 < Cohen’s d < 0.36). In longitudinal models, Asians reported slightly higher appetite loss, diarrhea, and financial difficulties than non-Asians (R^2^ increment < 0.0005). There was negligible uniform and non-uniform DIF, i.e., R^2^ 0 to 0.018, far below Zumbo’s (1999) criterion of 0.13. On average there were larger differences from norms for Asians (mean = 0.05, sd = 0.44) than non-Asians (mean = -0.07, sd = 0.36), but the size and direction of the differences varied considerably by domain, age, and gender.

**Conclusion:**

When compared to norms, Asian patients showed no systematic biases. DIF results supported this finding. We conclude that Asian ethnicity does not impact interpretation of EORTC QLQ-C30 scores.

**Supplementary Information:**

The online version contains supplementary material available at 10.1186/s12955-021-01860-3.

## Introduction

The use of patient-reported outcomes (PRO) has amplified the patient’s voice in clinical research [1]. While objective indices of health can be very relevant for assessing the impact of new treatments, they do not tell the whole story. Only the person experiencing the illness and the treatment can evaluate their impact on day-to-day functioning and internal symptom experience [2]. With the growth in the field of quality-of-life (QOL) research over the past three decades, there are often many options for measuring PROs for relatively common conditions. For rare conditions, however, the choice is more limited. While it is possible to craft measures using items from selected domains developed by the prominent Patient-Reported Outcome Measurement and Information System (PROMIS) [3] and the NeuroQOL systems [4], these systems may lack the language translations needed for international clinical trials, and translation requirements can be arduous and demanding. Developing and validating wholly new measures for rare conditions can be time-consuming and expensive, particularly because the validation process is iterative and federal regulatory agencies have high bars for accepting a PRO’s validity for a particular context [5].

It is thus not uncommon for clinical researchers focusing on rare conditions to use well-known and well-characterized legacy measures. With the benefit of time and seniority, legacy measures can be advantageous. They often have normative values across age, gender, region, and diagnosis, which facilitate interpretation within a study and comparison across studies. They may, however, lack normative values for a particular population. The present work focuses on the implementation of a widely-used cancer measure with a non-cancer rare disease.

Paroxysmal nocturnal hemoglobinuria (PNH) is a rare, life-threatening disease resulting in intravascular hemolysis and thrombosis with significant morbidity and premature mortality. With a prevalence of 12–13 out of one million people [6], PNH is characterized by uncontrolled activation of the terminal complement pathway leading to intravascular hemolysis and thrombosis [7–10], and it adversely affects QOL with significant morbidity and premature mortality if untreated [11–18]. People with PNH may experience a range of signs or symptoms across bodily systems, including hemoglobinuria, thrombosis, reduced kidney function, abdominal pain, pulmonary hypertension, chest pain, dyspnea, erectile dysfunction in males, end organ damage, and severe fatigue [12, 13, 19–22].

Clinical trials testing new treatment options for this condition rely on recruitment across many continents and countries. Such trials have relied on well-known legacy cancer measures, including the European Organisation for Research and Treatment of Cancer (EORTC) QLQ-C30 [23]. While the EORTC has available normative information for many European and North American countries, none exist for Asian countries [24, 25]. Normative data for Asian people are not currently available. The PNH clinical trials evaluating ravulizumab contained over 400 people, of whom about 50% were of Asian ethnicity [23, 26]. Thus, in order to compare treatments across participants, it would be important to know, first, whether Asian and non-Asian patients have similar scale/subscale mean scores.

In addition to investigating comparable expected values (i.e., mean scores), one would want to examine whether there is evidence of measurement non-invariance or differential item functioning (DIF) [27]. This important step would assess whether people in different ethnic groups respond differently to specific items by being more or less likely to endorse a given item in light of their scale/subscale score. These underlying differences in item response could confound interpretation. If DIF were found, then one could create ethnic-group-specific scoring that adjusts for such measurement non-invariance.

The present work thus aimed to compare Asian and non-Asian people with PNH on mean scores on the EORTC function and symptom scales and to investigate DIF by ethnicity in the PNH samples. Our null hypothesis would be that there are no differences in mean scores or item response by Asian ethnicity.

## Methods

### Sample

This secondary analysis utilized data from two PNH clinical trials (n = 441). Both clinical trials were phase-3, open-label studies evaluating the non-inferiority of ravulizumab compared to eculizumab. Trial 301 (ALXN1210-PNH-301) was conducted in PNH patients naïve to complement inhibitors [23]. Trial 302 (ALXN1210-PNH-302) was conducted in PNH patients who were stable on eculizumab for at least six months and of whom half were randomized to switch to ravulizumab and the other half remained on eculizumab [26].

Data available for analysis included longitudinal follow-up from baseline through the randomized 26-week period and also the extension period from 27 to 52 weeks. During the extension period all participants received ravulizumab. (For complete details on trial inclusion and exclusion criteria and procedures see references [23, 26].) The trials were conducted in accordance with the provision of the Declaration of Helsinki, the International Conference on Harmonization guidelines for Good Clinical Practice, and applicable regulatory requirements. The trials were approved by the institutional review board at each participating institution. All the patients provided written informed consent before participation.

### Measures

The EORTC QLQ-C30 is a comprehensive cancer-specific measure that consists of 30 items covering five function subscales (physical, role, emotional, cognitive, and social); nine symptom subscales/items (fatigue, nausea/vomiting, pain, dyspnea, insomnia, appetite loss, constipation, diarrhea, and financial difficulties); and a global health status/QOL subscale [28, 29]. Higher scores on the function and global health status/QOL scales and lower scores on the symptom scales reflect better health/QOL. Of note, each individual item’s response options, except those for global health status/QOL, moved toward worsening health. In other words, all items other than global health status/QOL were worded such that higher endorsement reflected worsening health. This is specifically relevant for interpreting DIF analyses. Further details of the measure are provided elsewhere [24].

Demographic characteristics collected in the trial datasets were age, years since diagnosis, baseline body mass index, sex, and region. Clinical data included trial, lactate dehydrogenase (LDH) stratum (stratum 1: < 1.5 × upper limit of normal (ULN); stratum 2: 1.5–3 × ULN; stratum 3: > 3 × ULN); packed red blood cells transfusion history (pRBC) stratum (stratum 1: 0 unit pRBC; stratum 2: 1–14 units pRBC; stratum 3: > 14 units pRBC); and binary “flags” (indicators) showing presence/absence of immunosuppressant treatment, aplastic anemia, myelodysplastic syndrome, and bone marrow disorder [23, 26].

### Statistical analysis

#### Sample comparisons

T-tests and chi-square statistics were used to compare Asian and non-Asian study participants across the demographic and clinical variables. In addition to p-values, effect sizes (ES) were summarized using indicators of variance explained. Cohen’s criteria [30] for small, medium, and large ES were used to facilitate interpretation of differences (> 0.01, > 0.06, and > 0.14 variance explained, respectively).

Propensity Scores. In order to make the soundest comparisons between Asian and non-Asian groups of people with PNH, we created propensity scores, specifically to be used via the covariate-adjustment method [31]. Such scores would be used in subsequent multivariate models to adjust for clinical and demographic variables that might confound the variable of interest, Asian ethnicity. Logistic models predicted Asian vs. non-Asian ethnicity group (dependent variable) with the following variables: age, baseline body mass index, treatment infusion start date, sex, observed LDH stratum, observed pRBC stratum, immune-suppressant treatment flag, aplastic anemia flag, myelodysplastic syndrome flag, bone marrow disorder flag, and trial. Trial was included as a covariate in the propensity score model because patients entering trial 302 had been stable on eculizumab for at least six months and had well-controlled hemolysis prior to randomization. These study patients thus would have better QOL scores at baseline, which may confound subsequent analyses. Further, there were trial differences in proportion of participants who were Asian: 75% of the Asian patients in the sample were from Trial 301, and 25% from Trial 302 (Table 1).

**Table 1:** Demographics of PNH patients at baseline

Variable		Asian (n = 171		Non-Asian (n = 270)		Difference, Asian vs. Non-Asian			
		Mean	SD	Mean	SD	*t*	Chi-square	*p*-value	Variance explained
Age		47.13	15.25	46.13	14.97	0.68		0.50	0.001
Years Since Diagnosis		8.72	8.28	9.39	9.05	-0.77		0.44	0.001
Baseline BMI		23.72	3.86	25.93	4.64	-5.14		< .0005	0.059
		**No.**	**%**	**No.**	**%**				
Female		71	42%	138	51%		3.86	0.05	0.009
Region	Europe	5	3%	214	79%		346.74	< .0005	0.787
	Japan	47	27%	0	0%				
	Latin America	1	1%	21	8%				
	North America	9	5%	24	9%				
	Rest of Asia Pacific	109	64%	11	4%				
Other variables included in propensity score									
Trial							43.75	< .0005	0.099
301		129	75%	117	43%				
302		42	25%	153	57%				
Observed LDH category							44.23	< .0005	0.100
	LDH < 1.5xULN	42	25%	153	57%				
	LDH 1.5—< 3xULN	16	9%	18	7%				
	LDH > = 3xULN	113	66%	99	37%				
Observed pRBC stratum							49.22	< .0005	0.112
	Not measured	42	25%	153	57%				
	0 unit pRBC	24	14%	19	7%				
	1–14 units pRBC	74	43%	82	30%				
	> 14 units pRBC	31	18%	16	6%				
Immuno-suppressant treatment	57	33%	167	62%			34.07	.0005	0.077
Aplastic anemia		60	35%	92	34%		0.05	0.83	< .001
Myelodysplastic syndrome	8	5%	14	5%			0.06	0.81	< .001
Bone marrow disorder	66	39%	100	37%			0.11	0.74	< .001

### Missing-data imputation for propensity scores.

The type of propensity-score computation we chose was done with logistic rather than mixed models; accordingly, the models used listwise deletion. Consequently, many rows with incomplete sets of covariates would be left out of the propensity-score computation. We thus performed the computation in two stages. In the first stage, we used all 11 covariates and were able to include 95% of patients. In the second stage, we filled in any missing propensity scores by employing only those 10 covariates that were completely filled in or for which a blank could be treated as its own category rather than a missing. The end result was propensity scores for all 441 patients in the analytic dataset.

#### Multivariate models

Analysis of covariance (ANCOVA) [32] was used to evaluate the association of Asian ethnicity with baseline EORTC function and symptom scores, after adjusting for propensity scores. Mixed models [33] were used to evaluate the association of Asian ethnicity with longitudinal EORTC function and symptom scores, after adjusting for propensity scores. Cohen’s *d* [30] was used to facilitate interpretation of differences (small ES = 0.20–0.49; medium ES = 0.50–0.79; large ES ≥ 0.80) for ANCOVA models, and pseudo-R^2^ increment to variance explained was used for mixed models (i.e., small ES = 0.010–0.059; medium ES = 0.060–0.139; large ES ≥ 0.140).

#### DIF comparisons

DIF analyses [27, 34] were conducted on the 24 EORTC QLQ-C30 items belonging to scales with at least two items. In theory, when two groups have the same amount of a trait (e.g., cognitive functioning), the likelihood of endorsing an item designed to measure that trait should not differ across groups. This analytic approach tests the hypothesis that people in one group systematically endorse items differently than those in another group, adjusting for the individuals’ total score on the scale in question.

The DIF analyses used ordinal logistic regression and involved building three nested models:

*Model 1*: Logit[P(Y≤j)] = *α*_j_ + *b*_1_(Total Score);

*Model 2*: Logit[P(Y≤j)] = *α*_j_ + *b*_1_(Total Score) + *b*_2_(Group); and

*Model 3*: Logit[P(Y≤j)] = *α*_j_ + *b*_1_(Total Score) + *b*_2_(Group) + *b*_3_(Total Score * Group),

where P(Y ≤ j) represents the probability that *j* is the rating-scale response category,

*α*_j_ is a regression constant, and each *b* is a regression coefficient.^1^

The log-likelihood ratio test compares Model 1 versus 2, Model 2 versus 3, and Model 1 versus 3. *Uniform DIF* is characterized by b_2_ being significant and the log likelihood test comparing Models 1 and 2 being significant (i.e., there is a significant main effect for Group). *Non-uniform DIF* is characterized by b_3_ being significant and the log-likelihood test comparing Models 2 and 3 being significant (i.e., there is a significant Group-by-total-score interaction). *Uniform and Non-uniform DIF* is characterized by the log-likelihood test comparing Models 1 and 3 being significant.

DIF analyses compared scores on baseline data and, separately, with longitudinal data. Uniform DIF analysis results are expressed in terms of a particular group being “favored” on an item, meaning it is “easier” for that group to endorse poor health for that item. The use of the term “easier,” as compared to “harder,” reflects the centrality of the idea of *difficulty* in the analysis of item response. Greater item difficulty would mean a higher bar for endorsing a particular response option, given one’s total score on that domain. We relied on Zumbo’s [27] criterion that a pseudo-R^2^ change (i.e., between Models 1 and 2 for uniform DIF; between Models 2 and 3 for non-uniform DIF) of at least 13% was necessary to indicate substantial DIF. Odds ratios were also obtained to facilitate interpretation. If significant, this type of DIF would reflect that Asians with PNH were responding differently to the EORTC items as compared to non-Asians with PNH.

SPSS Release 27 [35] and Stata/IC 16.1 [36] were used for all analyses.

## Results

### Sample

The clinical-trial data used in this secondary analysis included 441 PNH patients of whom 171 were Asian and 270 non-Asian. Table 1 provides descriptive demographic information about the study participants. They were most commonly in their forties, with an average of about nine years since diagnosis, and Asians were similar to non-Asians on these variables. Compared to non- Asian patients in these two trials, there were slightly more females in the Asian patient subgroup. About one quarter of the Asians in the study were from Japan, 64% were from other parts of Asia or the Pacific (i.e., Australia, Korea, Malaysia, Singapore, Taiwan, Thailand, and Turkey), and just 9% total were from Europe, North America or Latin America.

Asians were different from non-Asians on a number of clinical variables (Table 1). They were more likely to be treatment-naïve (i.e., enrolled in trial 301 rather than 302). Related to this trial inclusion, they were more likely to be in the worst LDH stratum, more likely to have data on transfusion history and be in the higher two pRBC strata, and less likely to be on immuno-suppressive treatment. Asian participants had a lower mean baseline body mass index (23.7 vs. 25.9). There were no differences by ethnicity on flags for aplastic anemia, myelodysplastic syndrome, or bone marrow disorder.

### Propensity scores

Additional file 1: Table S1 shows parameter coefficients for the propensity score model and provides a Q-Q plot and standardized mean difference of the propensity scores by ethnic group. The calculation was largely driven by pRBC stratum, treatment infusion start date, sex, and baseline body mass index.

### Baseline multivariate models

Table 2 shows results of the baseline ANCOVA models evaluating the association between Asian ethnicity and baseline EORTC function and symptom scores, after adjusting for propensity scores. Asians with PNH reported better role and emotional functioning scores and worse constipation and diarrhea scores than non-Asians. These differences had small ESs.

**Table 2:** Results of baseline ANCOVA models with Asian Ethnicity predicting EORTC QLQ-C30 score (428 <  = N <  = 433)*

Subscale	B for Asian group	SE	p**	*Cohen's d*
Function scales *(higher is better)*				
GlobalHealthStatus/QOL	−1.11	2.31	0.63	−0.05
Physical functioning	3.09	2.03	0.13	0.16
Role functioning	5.86	2.91	**0.04**	0.22
Emotional functioning	5.66	2.49	**0.02**	0.25
Cognitive functioning	−3.05	2.45	0.22	−0.14
Social functioning	−1.57	2.86	0.58	−0.06
Symptom scales *(higher is worse)*				
Fatigue	−0.48	2.70	0.86	−0.02
﻿ Nausea and vomiting	0.81	1.50	0.59	0.06
﻿ Pain	0.74	2.66	0.78	0.03
﻿ Dyspnoea	−1.91	3.10	0.54	−0.07
﻿ Insomnia	−1.81	3.10	0.56	−0.06
﻿ Appetite loss	1.21	2.37	0.61	0.06
﻿ Constipation	5.21	2.24	**0.02**	0.25
﻿ Diarrhea	6.56	2.01	**0.00**	0.36
﻿ Financial difficulties	4.98	3.29	0.13	0.17

^*^Adjusted for propensity score. **Values < 0.05 are in bold

### Longitudinal multivariate models

Table 3 shows results of the mixed models evaluating the association between Asian ethnicity and EORTC function and symptom scores over time, after adjusting for propensity scores. Asian PNH patients reported worse appetite loss, diarrhea, and financial difficulties than non-Asians, and no differences over time on function scores. These differences did not qualify as small ESs according to Cohen’s criteria [30].

**Table 3:** Results of mixed models with Asian Ethnicity predicting EORTC QLQ-C30 score*

Subscale	b	SE	p**	Pseudo-R^2^ increment
Function scales *(higher is better)*				
GlobalHealthStatus/QOL	−1.29	1.71	0.45	−0.0001
Physical functioning	1.07	1.51	0.48	0.0000
Role functioning	2.80	1.90	0.14	−0.0001
Emotional functioning	2.55	1.78	0.15	0.0000
Cognitive functioning	−2.25	1.81	0.21	0.0001
Social functioning	−1.80	1.95	0.36	0.0001
Symptom scales *(higher is worse)*				
Fatigue	0.45	1.94	0.82	0.0000
Nausea and vomiting	0.61	0.61	0.32	−0.0001
Pain	0.63	1.49	0.67	0.0000
Dyspnoea	−2.87	2.02	0.15	−0.0002
Insomnia	−1.67	2.13	0.43	−0.0002
Appetite loss	2.98	1.27	**0.02**	−0.0005
Constipation	3.06	1.61	0.06	0.0004
Diarrhea	3.00	1.25	**0.02**	0.0003
Financial difficulties	4.93	2.32	**0.03**	0.0002

^*^Adjusted for propensity score.  **Values < 0.05 are in bold

### Baseline DIF comparisons

Table 4 shows results of the baseline DIF comparisons. Negligible uniform DIF was detected in six function and three symptom items, but this evidence of DIF was evenly distributed within scales as favoring Asians and non−Asians, effectively cancelling out the effects. One physical function item showed evidence of negligible non-uniform DIF. Six items showed evidence of negligible uniform and non-uniform DIF. In all cases, the magnitude of the change in pseudo-R^2^ accounted for by the group effect and the group-by-total score interaction effect (i.e., variables used to identify DIF) was substantially smaller than Zumbo’s criterion of 13%. Thus, no notable DIF by ethnicity was detected in the baseline comparisons.

**Table 4:** DIF analyses by Asian: baseline data* **

Item no	Item content	Domain	Test of uniform DIF						Test of non-uniform DIF				Test of Uniform and non-uniform DIF			
			LR Chi^2^	P-value	Change in R^2^	DIF size	"Favored" group	Odds Ratio	LR Chi^2^	P-value	Change in R^2^	DIF size	LR Chi^2^	P-value	Change in R^2^	DIF size
eortc29	29. How would you rate your overall health during the past week?	Global	5.56	0.018	0.004	Negligible	Non-Asian	0.51	NS	NS	NS	NS	NS	NS	NS	NS
eortc30	30. How would you rate your overall quality of life during the past week?	Global	4.94	0.026	0.003	Negligible	Asian	1.90	NS	NS	NS	NS	NS	NS	NS	NS
eortc01	1. Do you have any trouble doing strenuous activities, like carrying a heavy shopping bag or a suitcase?	Physical	6.04	0.014	0.006	Negligible	Non-Asian	0.56	NS	NS	NS	NS	7.47	0.024	0.007	Negligible
eortc02	2. Do you have any trouble taking a long walk?	Physical	6.99	0.008	0.006	Negligible	Asian	1.91	NS	NS	NS	NS	9.01	0.011	0.008	Negligible
eortc03	3. Do you have any trouble taking a short walk outside of the house?	Physical	NS	NS	NS	NS	NS	NS	6.29	0.012	0.010	Negligible	6.75	0.034	0.011	Negligible
eortc04	4. Do you need to stay in bed or a chair during the day?	Physical	NS	NS	NS	NS	NS	NS	NS	NS	NS	NS	NS	NS	NS	NS
eortc05	5. Do you need help with eating, dressing, washing yourself or using the toilet?	Physical	NS	NS	NS	NS	NS	NS	NS	NS	NS	NS	NS	NS	NS	NS
eortc06	6. Were you limited in doing either your work or other daily activities?	Role	NS	NS	NS	NS	NS	NS	NS	NS	NS	NS	NS	NS	NS	NS
eortc07	7. Were you limited in pursuing your hobbies or other leisure time activities?	Role	NS	NS	NS	NS	NS	NS	NS	NS	NS	NS	NS	NS	NS	NS
eortc21	21. Did you feel tense?	Emotional	7.10	0.008	0.008	Negligible	Non-Asian	0.49	NS	NS	NS	NS	10.28	0.006	0.012	Negligible
eortc22	22. Did you worry?	Emotional	NS	NS	NS	NS	NS	NS	NS	NS	NS	NS	NS	NS	NS	NS
eortc23	23. Did you feel irritable?	Emotional	NS	NS	NS	NS	NS	NS	NS	NS	NS	NS	NS	NS	NS	NS
eortc24	24. Did you feel depressed?	Emotional	9.83	0.002	0.011	Negligible	Asian	2.26	NS	NS	NS	NS	13.57	0.001	0.015	Negligible
eortc20	20. Have you had difficulty in concentrating on things, like reading a newspaper or watching television?	Cognitive	NS	NS	NS	NS	NS	NS	NS	NS	NS	NS	NS	NS	NS	NS
eortc25	25. Have you had difficulty remembering things?	Cognitive	NS	NS	NS	NS	NS	NS	NS	NS	NS	NS	NS	NS	NS	NS
eortc26	26. Has your physical condition or medical treatment interfered with your family life?	Social	NS	NS	NS	NS	NS	NS	NS	NS	NS	NS	NS	NS	NS	NS
eortc27	27. Has your physical condition or medical treatment interfered with your social activities?	Social	NS	NS	NS	NS	NS	NS	NS	NS	NS	NS	NS	NS	NS	NS
eortc10	10. Did you need to rest?	Fatigue	NS	NS	NS	NS	NS	NS	NS	NS	NS	NS	NS	NS	NS	NS
eortc12	12. Have you felt weak?	Fatigue	14.76	0.000	0.014	Negligible	Asian	2.84	NS	NS	NS	NS	14.84	0.001	0.014	Negligible
eortc18	18. Were you tired?	Fatigue	4.77	0.029	0.005	Negligible	Non-Asian	0.54	NS	NS	NS	NS	NS	NS	NS	NS
eortc14	14. Have you felt nauseated?	Nausea	NS	NS	NS	NS	NS	NS	NS	NS	NS	NS	NS	NS	NS	NS
eortc15	15. Have you vomited?	Nausea	4.41	0.036	0.018	Negligible	Asian	3.52	NS	NS	NS	NS	NS	NS	NS	NS
eortc09	9. Have you had pain?	Pain	NS	NS	NS	NS	NS	NS	NS	NS	NS	NS	NS	NS	NS	NS
eortc19	19. Did pain interfere with your daily activities?	Pain	NS	NS	NS	NS	NS	NS	NS	NS	NS	NS	NS	NS	NS	NS

*NS = *Not significant*, **"R^2^*"* *refers to pseudo-R*^2^

### Longitudinal DIF comparisons

Table 5 shows results of the longitudinal comparisons. Uniform DIF was detected in six function and two symptom items, but again this evidence of DIF was evenly distributed within scales as favoring Asians and non-Asians, effectively cancelling out the effects. Non-uniform DIF was detected in three of the four emotional function items. For “tense” and “irritable”, Asians were less likely to endorse poor health at moderate emotional health. In contrast, for “depressed”, Asians were more likely to endorse poor health at low and moderate levels of emotional health. In other words, Asians with PNH who had a total score indicative of moderate Emotional Functioning were less likely to endorse being tense or irritable and more likely to endorse being depressed. These different non-uniform DIF directions would have a negligible impact.

**Table 5:** DIF Analyses by Asian: longitudinal data

Item no	Item content	Domain	Test of uniform DIF			Test of non-uniform DIF
			P-value (group effect)	"Favored" group	Odds Ratio	P-value (on Interaction term)
eortc29	29. How would you rate your overall health during the past week?	Global	NS	NS	NS	NS
eortc30	30. How would you rate your overall quality of life during the past week?	Global	NS	NS	NS	NS
eortc01	1. Do you have any trouble doing strenuous activities, like carrying a heavy shopping bag or a suitcase?	Physical	p = .001	Non-Asian	0.43	NS
eortc02	2. Do you have any trouble taking a long walk?	Physical	p = .005	Asian	2.13	NS
eortc03	3. Do you have any trouble taking a short walk outside of the house?	Physical	NS	NS	NS	NS
eortc04	4. Do you need to stay in bed or a chair during the day?	Physical	NS	NS	NS	NS
eortc05	5. Do you need help with eating, dressing, washing yourself or using the toilet?	Physical	NS	NS	NS	NS
eortc06	6. Were you limited in doing either your work or other daily activities?	Role	NS	NS	NS	NS
eortc07	7. Were you limited in pursuing your hobbies or other leisure time activities?	Role	NS	NS	NS	NS
eortc21	21. Did you feel tense?	Emotional	p = .001	Non-Asian	0.44	p = .011
eortc22	22. Did you worry?	Emotional	NS	NS	NS	NS
eortc23	23. Did you feel irritable?	Emotional	NS	NS	NS	p = .035
eortc24	24. Did you feel depressed?	Emotional	p < .001	Asian	3.21	p < .001
eortc20	20. Have you had difficulty in concentrating on things, like reading a newspaper or watching television?	Cognitive	p = .001	Non-Asian	0.36	NS
eortc25	25. Have you had difficulty remembering things?	Cognitive	p < .001	Asian	3.53	NS
eortc26	26. Has your physical condition or medical treatment interfered with your family life?	Social	NS	NS	NS	NS
eortc27	27. Has your physical condition or medical treatment interfered with your social activities?	Social	NS	NS	NS	NS
eortc10	10. Did you need to rest?	Fatigue	NS	NS	NS	NS
eortc12	12. Have you felt weak?	Fatigue	p = .002	Asian	2.20	NS
eortc18	18. Were you tired?	Fatigue	p = .001	Non-Asian	0.50	NS
eortc14	14. Have you felt nauseated?	Nausea	NS	NS	NS	NS
eortc15	15. Have you vomited?	Nausea	NS	NS	NS	NS
eortc09	9. Have you had pain?	Pain	NS	NS	NS	NS
eortc19	19. Did pain interfere with your daily activities?	Pain	NS	NS	NS	NS

*NS* = *Not significant*

## Discussion

To our knowledge, this is the first study to address Asian ethnicity and item response on the EORTC. The present study found small ES differences in EORTC scale scores between Asians and non-Asians at baseline and over time, suggestive of slightly better role and emotional functioning at baseline and slightly worse appetite loss, diarrhea, and financial difficulties over time. These analyses adjusted for differences in demographic and clinical risk factors, and they revealed at most small differences at baseline and negligible differences over time. Small effect sizes do not generally meet the threshold for “clinical significance” [37]. Further, only negligible DIF effects were detected at baseline or over time, and these effects did not systematically “favor” one ethnic group over the other. Thus, while there were some ethnicity-related differences, they do not appear to reflect a systematic bias in EORTC scores or item response.

These results would suggest that interpreting differences in Asian versus non-Asian people with PNH is not confounded with differences in item functioning across groups. In general, after accounting for differences in total functional problems and symptom burdens, respectively, no one ethnic group was more likely to endorse specific functional problems or symptom burdens. Accordingly, digestive symptoms at baseline seem more problematic to Asians, and for them over time such symptoms and financial difficulties are, negligibly, more problematic. Such digestive-symptom differences may be worthy of targeted intervention for symptom relief. In contrast, the finding that Asians reported better role and emotional functioning at baseline was not true over the full course of follow-up, so it may not have much clinical importance.

While the study has the advantage of a relatively large sample of this rare condition across diverse geographic regions, the limitations must be acknowledged. First, there is limited demographic information about trial participants, and so testing hypotheses about other variables that might be responsible for functioning or symptom-burden differences is not possible. For example, information on cultural characteristics is not known. The relationship between ethnicity and QOL outcomes could be mediated or moderated by health behaviors (e.g., smoking, exercise, dietary habits), or socioeconomic status (e.g., employment status, education level, marital status). Such limitations are common disadvantages in secondary data analysis. Second, six of the nine scales included only two survey items, and therefore, findings from the DIF analyses should be interpreted with some caution.

### Conclusions

In summary, we did not find evidence of systematic differences or biases between Asian and non-Asian patients with PNH. We conclude that Asian ethnicity is not likely to impact interpretation of EORTC QLQ-C30 scores.

## Supplementary Information

**Additional file 1.** More Information about the Propensity Score.

## Data Availability

Alexion will consider requests for disclosure of clinical study participant-level data provided that participant privacy is assured through methods like data de-identification, pseudonymization, or anonymization (as required by applicable law), and if such disclosure was included in the relevant study informed consent form or similar documentation. Qualified academic investigators may request participant-level clinical data and supporting documents (statistical analysis plan and protocol) pertaining to Alexion-sponsored studies. Further details regarding data availability and instructions for requesting information are available in the Alexion Clinical Trials Disclosure and Transparency Policy at https://alexion.com/our-research/research-and-development.

## Abbreviations

DIF: Differential item functioning
EORTC QLQ-C30: European Organisation for Research and Treatment of Cancer Quality of Life Questionnaire-C30 measure
ANCOVA: Analysis of covariance
MANCOVA: Multivariate analysis of covariance
PNH: Paroxysmal nocturnal hemoglobinuria
PRO: Patient-reported outcome
QOL: Quality of life

## Footnotes

1 *Technical note: The standard ordinal logistic regression model parameterization indicates the probability of moving to a lower category for each unit increase in X. Stata transforms the output such that positive coefficients indicate higher probabilities of moving to a “higher” category for each unit increase in X, easing interpretation.*

## Code availability

Requests for software code will be considered, and if they are deemed reasonable code will be made available.
