# Supplementary material for: No impact of Asian ethnicity on EORTC QLQ-C30 scores: Group differences and differential item functioning in paroxysmal nocturnal hemoglobinuria
Source: Health Qual Life Outcomes. 2021 Sep 28;19:228. doi: 10.1186/s12955-021-01860-3 (PMC8477512; doi:10.1186/s12955-021-01860-3)
Supplement: Supplementary file 1 — Additional file 1. More Information about the Propensity Score. [file 12955_2021_1860_MOESM1_ESM.docx]

**Supplemental File 1: More Information about the Propensity Score**

Standardized Mean Difference between Asian and non-Asian patients’ Propensity Score: 0.89

Q-Q plot for Propensity Score:


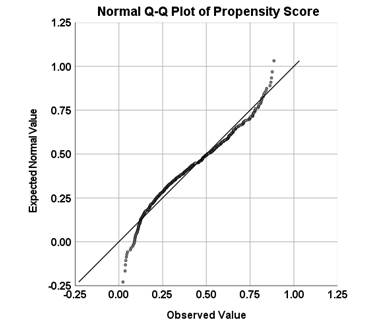


Histogram of Propensity Score


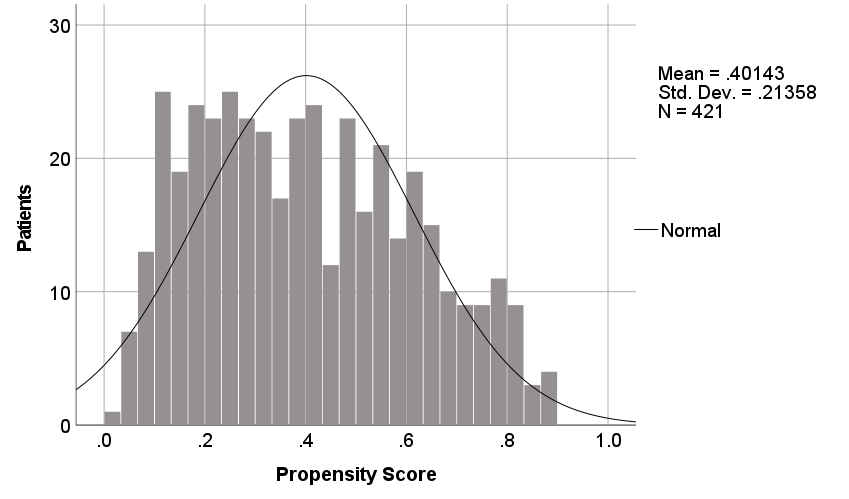


It should be noted that ANCOVA predictors, like GLM predictors generally, need not follow normal distributions the way model residuals need to.
